# Comparison of the efficacy between conventional moxibustion and smoke-free moxibustion on knee osteoarthritis: study protocol of a randomized controlled trial

**DOI:** 10.1186/s13063-017-1846-2

**Published:** 2017-04-24

**Authors:** Lin-lin Zhu, Jian-ying Zhou, Ling Luo, Xiang Wang, Jia-xi Peng, Sha-sha Chen, Hai-Yan Yin, Qiao-Feng Wu, Cheng-shun Zhang, Peng Lv, Yong Tang, Shu-guang Yu

**Affiliations:** Chengdu University of Traditional Chinese Medicine Chengdu, Sichuan, China

**Keywords:** Moxa smoke, Moxibustion, Knee osteoarthritis, Randomized controlled trial, Protocol

## Abstract

**Background:**

Conventional moxibustion is a representative non-drug intervention in traditional Chinese medicine, and it has been reported to produce encouraging results and benefits in relieving symptoms and improving the quality of life for patients with knee osteoarthritis (KOA) in previous clinical trials and systematic reviews. Given that increasing concerns on the safety of generated smoke from conventional moxibustion have received much attention, smoke-free moxibustion is regarded as a potential alternative. However, whether smoke-free moxibustion would display a similar efficacy to that of conventional moxibustion still remains unclear. Therefore, this randomized controlled trial attempts to investigate the difference of efficacy between conventional moxibustion and smoke-free moxibustion in patients with KOA.

**Methods/design:**

This is a multicenter, randomized, single-blinded, parallel-group clinical trial. A total of 138 eligible participants with KOA will be randomly allocated to two groups (conventional moxibustion group and smoke-free moxibustion group) in seven hospitals in China. Participants will receive 12 sessions of moxibustion treatment at three acupoints (EX-LE4, ST35, and ST36) over a period of 4 weeks (3 sessions per week). A smoke-removing device is placed at the top of the moxibustion device for the smoke-free moxibustion group (*n* = 69), while the conventional moxibustion group (*n* = 69) is treated with traditional moxibustion. The primary outcome measure will be the change of the global scale of the Western Ontario and McMaster Universities Osteoarthritis Index (WOMAC) from the baseline to 4 weeks. Secondary outcomes include the visual analog scale VASand Patient Global Assessment scores. Follow-up measurements will be performed on the 8th and 12th weeks after random allocation.

**Discussion:**

This study will contribute to providing a solid foundation for the selection of moxibustion in clinical application as well as future research in moxibustion therapy.

**Trial registration:**

ClinicalTrials.gov, NCT02772055. Registered on 12 May 2016.

**Electronic supplementary material:**

The online version of this article (doi:10.1186/s13063-017-1846-2) contains supplementary material, which is available to authorized users.

## Background

Knee osteoarthritis (KOA) is the most common form of arthritis and is a major cause of disability and limitation of activity, especially in elderly patients [1]. It is characterized by pain and functional limitations, leading to a reduction in quality of life [2]. Osteoarthritis of the knee makes a significant impact on society, with more than 20 million Americans and 35 to 40 million Europeans developing this condition in their lifetime [3, 4]. Some recent international guidelines advocate non-pharmacological care for patients with knee pain [5–7]. As a representative non-drug intervention in traditional Chinese medicine (TCM) and a form of acupuncture [8–10], moxibustion has been reported to produce encouraging results and benefits in relieving symptoms and improving the quality of life for patients with KOA in many studies, including clinical trials and systematic reviews [11–15].

The mechanism of moxibustion is mainly related to its thermal effects, radiation effects, the pharmacological activity of moxa, and its combustion products (volatile oil, brown tar-like substances, and moxa smoke). Moxa smoke is a primary combustion product of moxibustion, and its potential effect on the health and environment may be controversial [16]. It was reported that moxa smoke has antibacterial and antiviral effects effective in treating various conditions including wound infections, vaginal itching, uterine prolapse, anal fistulas, common warts, and so forth [17–22]. However, some studies demonstrated that high levels of monoaromatic hydrocarbons, formaldehyde, and polycyclic aromatic hydrocarbons in the moxibustion room may have adverse effects on human health [23–25]. Epidemiological investigation also showed that the moxa smoke induced a variety of undesirable reactions such as dry throat, dry eye, and coughing [26]. Furthermore, with their increased environmental awareness, people have started to question the safety of the smoke produced by burning moxa [27–29]. Because of concerns as to the potential toxicity of the smoke, many acupuncturists prefer to use a smoke purification device to remove moxa smoke during the moxibustion treatment, but few studies have reported the difference of clinical efficacy between conventional moxibustion and smoke-free moxibustion (moxibustion using the smoke-removing device).

Thus, we are currently conducting a randomized controlled clinical trial to compare the efficacy of conventional moxibustion and smoke-free moxibustion for patients with KOA.

## Methods/design

### Design

**Fig. 1:**
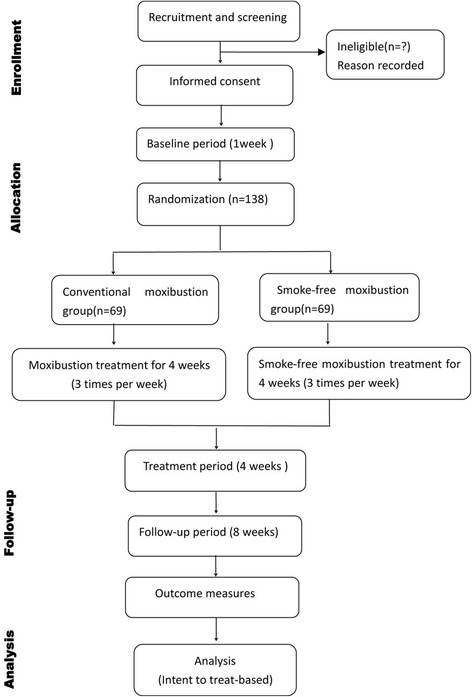
Flowchart of the study process. Participants with a diagnosis of KOA will be recruited from seven centers. All project general practitioners (*GPs*) were trained for 1 day. The trial period will consist of 12 sessions of moxibustion treatment over a period of 4 weeks. The treatment frequency will be three sessions per week, and there will be a 2-month follow-up period. The outcomes will be measured at every visit

This is a multicenter, randomized, single-blinded, parallel-group design clinical trial conforming to Consolidated Standards of Reporting Trials (CONSORT) and Standards for Reporting Interventions in Clinical Trials of Acupuncture (STRICTA) guidelines [30, 31]. A Standard Protocol Items: Recommendations for Interventional Trials (SPIRIT) checklist is provided in Additional file 1. A total of 138 eligible participants with KOA will be randomly allocated into two groups (moxibustion group or smoke-free moxibustion group) with a 1:1 allocation ratio. Eligible participants who meet the clinical criteria for KOA formulated by the American College of Rheumatology (ACR) will be enrolled in this trial [32]. Seven clinical research centers in China will participate in this trial: Central Hospital of ZiBo, Chengdu First People’s Hospital, Sichuan Second Hospital of TCM, Pi County People’s Hospital, Xinjin County TCM Hospital, Qionglai TCM Hospital, and Nanjing Hospital of TCM. The study period is 13 weeks, with a 1-week baseline period, 4-week treatment phase, and 8-week follow-up phase. After randomization, eligible participants will receive 12 sessions of moxibustion treatment over a period of 4 weeks. Patients will be assessed at the baseline visit, as well as at 2 weeks, 4 weeks, 8 weeks, and 12 weeks after allocation. The trial diagram demonstrates the procedures of the study (see Fig. 1). Table 1 outlines the measures used throughout this trial.

**Table 1:** Study design schedule

Period	Inclusion	Treatment		Follow-up	
	Baseline	First assessment	Second assessment	Third assessment	Fourth assessment
	0 week	2 weeks after inclusion	4 weeks after inclusion	8 weeks after inclusion	12 weeks after inclusion
Inclusion confirmed	√				
Informed consent	√				
Randomization and blinding	√				
Sociodemographic characteristics	√				
Disease history	√		√		
Treatment history	√		√		
Comorbidity	√		√	√	√
Current treatment	√		√	√	√
Outcomes					
WOMAC	√	√	√	√	√
VAS	√	√	√	√	√
PGA		√	√	√	√
Trial evaluation					
Outcome analysis					√
Adverse event			√		
Causes of dropout			√		
Safety analysis			√		
Compliance analysis			√		

*WOMAC* Western Ontario and McMaster Universities Osteoarthritis Index, *VAS* visual analog scale, *PGA* Patient Global Assessment

### Inclusion criteria

Participants meeting the following criteria will be included:

- Aged between 40 and 75 years
- Diagnosed with KOA formulated by the American College of Rheumatology (ACR)
- Average severity of knee pain no more than 7 points on a visual analog scale (VAS) for most days during the past month
- Agreed to not use paregoric during the whole treatment phase
- Willingness to participate in a randomized study and to sign the informed consent form.

### Exclusion criteria

Participants will be excluded if they have any of the following conditions:

- Pain in the knee that may be caused by inflammatory, malignant, or autoimmune disease or by traumatic injury
- Serious diseases including cancer, uncontrolled hypertension, diabetes mellitus requiring insulin injection, life-threatening cardiovascular or neurological events, chronic respiratory disease, a hemorrhagic disorder, or serious mental diseases
- Knee replacement surgery, arthroscopy of the affected knee within the past year, steroid or hyaluronic acid injection in the knee joints within the previous 3 months
- Life-threatening cardiovascular or neurological events within the past year
- Physiotherapy or other treatments for osteoarthritis knee pain (with the exception of non-steroidal anti-inflammatory drugs) during the previous 4 weeks
- Current participation in another clinical trial
- Accepted acupuncture, moxibustion, cupping, or herbal medicine within the past 4 weeks.

### Sample size calculation

A power analysis has been undertaken before conducting this trial for further calculation. The significance level is 0.05, and the statistical power is 0.90, consistent with a previous trial on moxibustion for KOA and our pilot study [33]. To compensate for an anticipated dropout rate of 15%, 138 patients with KOA (69 in each group) will be recruited from seven centers in China.

### Recruitment strategies

There will be three strategies for KOA participant recruitment. First, participants will be recruited from the outpatient and inpatient departments of the seven hospitals. Second, printed recruitment posters will be distributed to local communities. Finally, advertisements will be posted in local newspapers and through the website.

### Random assignment

Each eligible subject will be randomly assigned to either the conventional moxibustion group or the smoke-free moxibustion group. A random number list will be generated with SPSS 16.0 (SPSS Inc., Chicago, IL, USA) by a statistician who is not directly involved with the study. The researchers in each clinical research center will call the independent statistician at the center for the randomized number, and the statistician will be immediately informed of the participant’s randomized number and treatment group by mobile message. The independent researcher will record the patient’s details and the number assigned to that patient. Central randomization has strict limits of authority: no one can check the files except the top principal investigator.

### Blinding

This is a single-blinded trial; participants are unaware of the assignments. Participants will be separated and treated in two different rooms. Meanwhile, the assessors of therapeutic effect, the data manager, and the statistician will be kept blinded to the treatment allocation. To prevent accidental un-blinding, there will be no contact between the study team and the subjects until after the data cleaning, database lockdown, and analysis are complete.

### Interventions

**Fig. 2:**
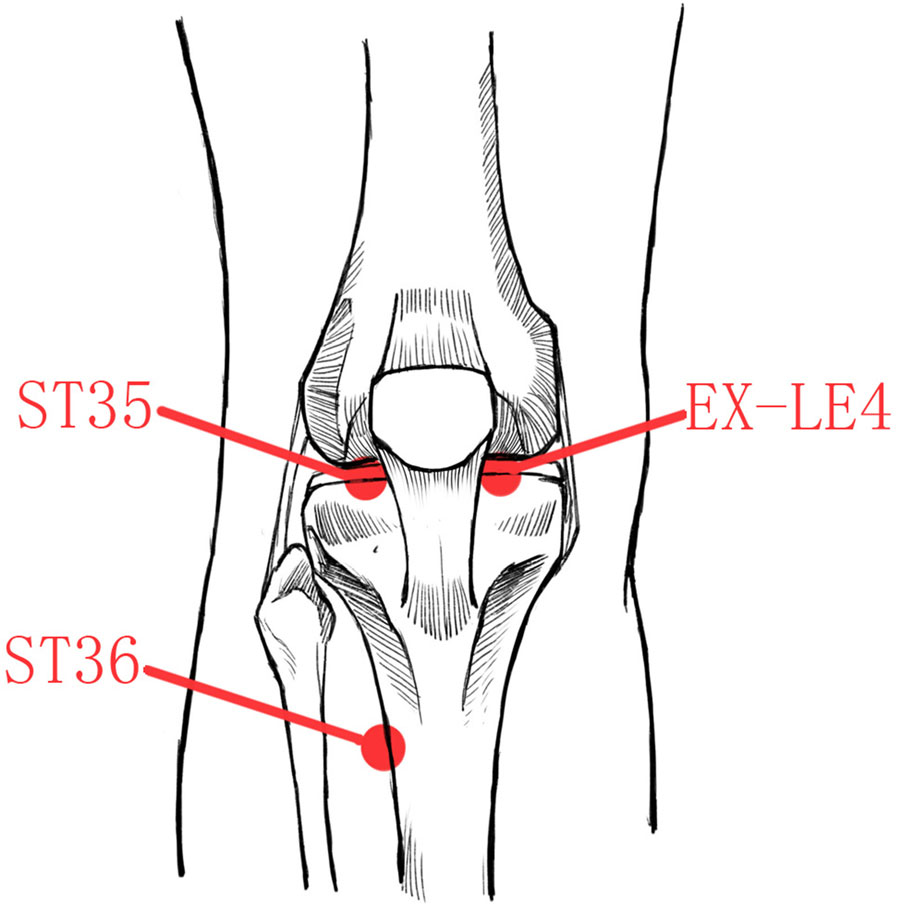
Acupoints used in the trial. The lines refer to the acupoints Dubi (ST35) and Zusanli (ST36) on the lateral side of the knee and Neixiyan (EX-LE4) on the medial side of the knee

The treatment will be applied in 12 sessions, three times per week for 4 weeks. The conventional moxibustion group and the smoke-free moxibustion group will receive moxibustion at three standard acupuncture points: Neixiyan (EX-LE4), Dubi (ST35), and Zusanli (ST36) [11, 33] (Fig. 2). The selection of acupoints is based on TCM meridian theory to treat knee joint pain, known as the “Bi syndrome,” and on some similar studies [11, 12, 34–36]. All project general practitioners (GPs) have completed at least 5 years of training in acupuncture and moxibustion and are registered as Chinese medicine practitioners by the National Health and Family Planning Commission of the People’s Republic of China. Project GPs were trained for 1 day by an investigator who is an experienced medical acupuncturist, to standardize all aspects of the treatment protocol. To ensure implementation of the study, the investigator must maintain regular contact with the GPs to answer and settle any treatment-related problems. Each GP was provided with a detailed treatment manual describing administration of the moxibustion interventions as well as the trial protocol. Two separated moxibustion rooms were provided for the different groups by the centers, doors and windows will be opened in the treatment process. The participants are usually required to wear loose clothing to expose the knee joints and to maintain a comfortable sitting position for treatment.

### Moxibustion group (group A)

**Fig. 3:**
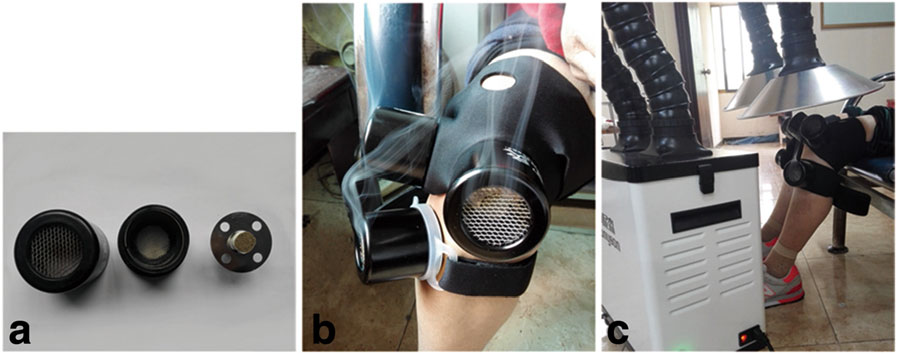
Diagram of the moxibustion and smoke-free moxibustion devices. **a** This new moxa device is composed of three parts. **b** Participants are treated at acupoints Dubi (ST35), Zusanli (ST36), and Neixiyan (EX-LE4) with devices consisting of a moxa pillar. The moxa device has an opening in the center that allows heat and smoke from the burning pillar to stimulate the acupoints. **c** In the smoke-free moxibustion group, investigators place a purification device at the top of the moxibustion device to remove the moxa smoke

The moxibustion group will use a moxa device (Yijiu moxa device, Maanshan, Anhui, China) at the acupoints (EX-LE4, ST35, ST36). The moxa pillar is cylindrical, 1.5 cm in diameter, and 3 cm long. A lighted moxa pillar will be put into the moxa device attached at each acupoint, and the therapist will make sure the patient feels a warm but not scorching sensation (Fig. 3). One acupoint is treated for 30 min each time. Three acupoints are treated by moxibustion at the same time.

### Smoke-free moxibustion group (group B)

In contrast to the moxibustion group, a specifically designed device (Shenzhen Conyson Electronic Technology Co, Ltd., C200 moxa smoke purification device, Shenzhen, China) is applied to remove the moxa smoke for the smoke-free group in the process of moxibustion. Otherwise, the selection of acupoints and other interventions in both groups must be the same (Fig. 3).

### Outcome assessments

#### Primary outcome measurement

The primary outcome measure will be the mean change in the global scale value of the Western Ontario and McMaster Universities Osteoarthritis Index (WOMAC) from baseline to 4 weeks [37]. The WOMAC, one of the most widely used arthritis assessment tools, is composed of 24 questions to assess disability related to osteoarthritis. It includes three subscales to measure pain (5 questions), stiffness (2 questions), and physical function (17 questions) regarding KOA; higher scores indicate more severe impairment. All 24 questions will be listed in a numerical rating scale ranging from 0 (no symptoms) to 3 (maximum symptoms). If both knees are affected, the more severe knee will be assessed. Physicians who are blinded to the treatment allocation will help patients perform the self-assessment and collect and record the total score.

#### Secondary outcome measurements

The secondary outcomes include the mean changes in the VAS and Patient Global Assessment (PGA) scores. The pain intensity of KOA will be assessed using a 100 mm VAS (0, absence of pain; 100, the worst pain imaginable). The pain VAS is a standard tool in chronic pain studies for measuring pain intensity. We will evaluate the mean change in the VAS at baseline (0 week) and at 2, 4, 8, and 12 weeks after allocation. The PGA score is a self-reported 5-point measurement used to evaluate overall improvement after treatment. It has been used in various studies to assess therapeutic effects [38–40]. Participants individually evaluate their improvement by selecting one of 5 options (much improved, minimally improved, no change, minimally worse, or much worse) at 2, 4, 8, and 12 weeks from the baseline.

### KOA diary

Subjects will be provided with KOA diaries to record the specific time of knee pain attacks, the associated phenomena, and any medication used. This information will be recorded every day throughout the study period. All patients will be carefully instructed on how to use this diary to rate their symptoms.

### Statistical analysis plan

#### Data integrity

The research will ensure that no record will be missed or omitted in the original data source. Any corrections should be explained in the appended notes signed and dated by the physicians participating in the clinical trial. The primary input of the data is not permitted to be changed. After the observation of a clinical case, a case report form (CRF) will be submitted to the project directors for verification. The principal investigators will have full access to the data. The data manager will clarify any questions regarding the CRF with the researchers via clinical supervisors. Researchers should answer any questions as soon as possible so that the data manager will be able to conduct modification, validation, and input of the data. The data monitoring committee comprises a medical statistician and an epidemiologist, who are independent from the investigators and sponsors, for source data organizing, coding, and range checking for data values to ensure data quality.

### Analysis

The primary analysis of the data will be undertaken using the intention-to-treat principle with all randomized patients included in the analyses. A multiple imputation adjustment approach will be used for the missing data. Demographic and baseline data will be analyzed with standard, descriptive statistics. Between-group differences will be tested using repeated measure analyses of variance. The accepted level of significance for all analyses is *P* < 0.05. The whole data analysis process will be performed by statisticians who are independent from the research team and blinded to the group settings. SPSS software (SPSS 16.0, SPSS Inc., Chicago, IL, USA) will be used for the data analysis.

### Safety monitoring

Possible adverse events (AEs) resulting from moxibustion include blisters, redness, itching, and burns. All unexpected and unintended responses will be reported as AEs by the researcher at every visit. AEs will be carefully recorded in the CRF by the corresponding research staff.

## Discussion

Moxibustion is widely used in Asian countries to treat various disorders and is reported to be effective for relieving the pain and functional limitations of KOA [10, 12, 13]. Recent research has focused on the composition and the safety of moxa smoke, but few studies have considered the effect of moxibustion with removal of the moxa smoke [25]. The aim of this trial is to compare the effectiveness of conventional moxibustion and smoke-free moxibustion for patients with KOA.

In clinical application, the smoke-free moxa sticks have been used because of the concerns about moxa smoke; however, some experienced acupuncturists consider that its clinical efficacy may not be equal to that of conventional moxibustion. In the present study, a special device removing the moxa smoke will not be used in the conventional moxibustion group but only in the smoke-free group, and the same kind of moxa sticks will be chosen in these two groups to ensure that other conditions except for the moxa smoke reach consensus. The study aims to explore the influence on effectiveness of removing moxa smoke in the process of moxibustion. The device to remove the moxa smoke has been used in clinical practice, and our design is based on clinical application.

Because of the objective of this trial to clarify whether removing moxa smoke may be interfering with the clinical efficacy of conventional moxibustion, and the difficulty of using the double-blind methodology in acupuncture and moxibustion, we designed a single-blind study; therefore, it is not necessary to set up a wait-list group. The treatment acupoints and intervention period are based on some positive result studies and clinical experience in order to ensure their effectiveness [11–13, 15].

The design and methodological rigor of this trial will allow for collection of high-quality data to compare the effectiveness of conventional moxibustion and smoke-free moxibustion for patients with KOA. Thus, the trial will contribute to providing a selection of moxibustion in practice, as well as promote the clinical application of moxibustion therapy.

### Trial status

This study is currently in the recruitment phase. Participant recruitment started in June 2016 and is expected to end in May 2017.

## Additional file

Additional file 1: SPIRIT 2013 checklist: recommended items to address in a clinical trial protocol and related documents. (DOC 119 kb)

## Abbreviations

ACR: American College of Rheumatology
AE: Adverse event
CRF: Case report form
GP: General practitioner
KOA: Knee osteoarthritis
TCM: Traditional Chinese medicine
VAS: Visual analog scale
WOMAC: Western Ontario and McMaster Universities Osteoarthritis Index
